# Safety and efficacy of percutaneous nephrolithotomy for the treatment of paediatric urolithiasis

**DOI:** 10.1308/003588412X13373405387014

**Published:** 2012-05

**Authors:** R Veeratterapillay, MBK Shaw, R Williams, P Haslam, A Lall, M De la Hunt, ST Hasan, DJ Thomas

**Affiliations:** Freeman Hospital and Great North Children Hospital, Newcastle Upon Tyne,UK

**Keywords:** Percutaneous nephrolithotomy, Stones, Complications, Paediatrics

## Abstract

**INTRODUCTION:**

Paediatric percutaneous nephrolithotomy (PCNL) has revolutionised the treatment of paediatric nephrolithiasis. Paediatric PCNL has been performed using both adult and paediatric instruments. Stone clearance rates and complications vary according to the technique used and surgeon experience. We present our experience with PCNL using adult instruments and a 28Fr access tract for large renal calculi in children under 18 years.

**METHODS:**

All patients undergoing PCNL at our institution between 2000 and 2009 were reviewed. Demographics, surgical details and post-operative follow-up information were obtained to identify stone clearance rates and complications.

**RESULTS:**

PCNL was performed in 32 renal units in 31 patients (mean age: 10.8 years). The mean stone diameter was 19mm (range: 5–40mm). Twenty-six cases required single puncture and six required multiple tracts. Overall, 11 staghorn stones, 10 multiple calyceal stones and 11 single stones were treated. Twenty-seven patients (84%) were completely stone free following initial PCNL. Two cases had extracorporeal shock wave lithotripsy for residual fragments, giving an overall stone free rate of 91% following treatment. There was no significant bleeding or sepsis encountered either during the operation or in the post-operative setting. No patient required or received a blood transfusion.

**CONCLUSIONS:**

Paediatric PCNL can be performed safely with minimal morbidity using adult instruments for large stone burden, enabling rapid and complete stone clearance.

Paediatric urolithiasis is an uncommon condition that accounts for 0.13–0.94 cases per 1,000 hospital admissions in the Western world.^1^ There is evidence that urolithiasis is increasing in incidence.^2^ Most stones affect the upper urinary tract and many studies have shown a higher prevalence among male patients.^3,4^ Paediatric stone disease is often complex and related to underlying metabolic or renal anatomical abnormalities where high recurrence rates are seen.

The management of renal stones has changed over the past few decades from open surgery to a minimally invasive approach. Extracorporeal shock wave lithotripsy (ESWL) was pioneered in the 1980s and established itself rapidly as a good treatment option in children but it can be limited by patient compliance and may require general anaesthesia. ESWL has been shown to be effective, safe and to achieve good stone fragmentation rates but bulky stones can lead to large ureteric fragments that can be difficult to manage in children.^5–7^ Paediatric percutaneous nephrolithotomy (PCNL) was first described in 1985 and allows the treatment of patients with a larger stone burden or those in whom ESWL is contraindicated or unlikely to be successful.^8^

Paediatric PCNL has been performed using both adult instruments and paediatric instruments.^9,10^ The rationale for small instruments or ‘miniperc’ is to reduce morbidity while not compromising stone clearance rates. Stone clearance rates and complications vary according to the technique used and surgeon experience. We present our experience with PCNL using adult instruments for large renal calculi at a tertiary referral centre in the UK.

## Methods

The details of all patients under the age of 18 years having undergone PCNL between 2000 and 2009 were retrieved from a prospectively acquired departmental database. Demographic, surgical details and post-operative follow-up information were retrieved from a combination of the recorded database information and subsequent chart review.

Detailed information about the pre-operative nature of the treated stones was recorded, including size, location and multiplicity. In addition, surgical information was noted regarding the technique used to treat the stone and the immediate success rate. Follow-up information with regard to the most up-to-date stone status and the need for subsequent treatment of residual or recurrent stone was also recorded.

All PCNLs were performed to the same basic technique. Patients were treated prone under general anaesthesia, with intravenous prophylactic antibiotics given according to urine culture sensitivities. In all cases, a cystoscopy was performed with a 9Fr cystoscope with the patient supine in a modified Lloyd-Davies position. Retrograde pyelography was performed using a 6Fr Beacon® tipped catheter (Cook, Bloomington, IN, US) in order to delineate the anatomy of the renal collecting system. The Beacon® tipped catheter was left in situ (usually in the uppermost calyx) attached to a urethral catheter in order to allow the instillation of radiographic contrast during the percutaneous puncture.

The patient was turned prone on a pressure-relieving mattress with an inflatable rubber balloon placed under the upper abdomen to reduce renal movement. Percutaneous placement of an 18G two-part trocar needle (Cook, Bloomington, IN, US) was guided by a combination of ultrasonography and limited x-ray screening. Subsequent track formation was performed by serial, coaxial dilatation using Alken dilators to allow placement of a 28Fr Amplatz sheath.

In the majority of cases a single percutaneous track was sufficient to allow stone clearance but a second percutaneous track was required occasionally. The primary operating instrument was a Storz nephroscope, augmented as necessary by the use of a flexible cystoscope (16Fr) or flexible ureterorenoscope (8Fr). Stone removal was performed using graspers and ultrasound lithotripsy with integrated suction. A ballistic lithoclast or holmium lasertripsy was employed as required to fragment stones not responding to the other energy modalities. On completion of the procedure, either a 7Fr pigtail nephrostomy or antegrade Beacon® tipped catheter was left in situ in an antegrade fashion for 24 hours. In complex cases, an 18Fr medical grade silicon drain was placed down the track over the existing percutaneous access for 24 hours following surgery.

Post-operatively, drains were removed within 24 hours and the patients given oral analgesia. Early ambulation was encouraged. Patients had radiological assessment of stone clearance after surgery to plan follow-up. All patients were seen for outpatient review at six weeks and underwent either plain radiography or ultrasonography at that point dependent on the stone composition and visibility on pre-operative imaging. The continuation of outpatient follow-up and imaging was performed on a tailored, individual patient basis. The follow-up of patients was initially performed as joint care between the urologist and paediatric nephrologists. Once stone free, ongoing care was provided by the referring paediatric nephrologist.

## Results

PCNL was performed on 32 renal units in 31 patients with a mean age at the time of surgery of 10.8 years (range: 2.8–17.9 years) (Table 1). Surgery was performed on 17 girls and 14 boys. The mean stone diameter was 19mm (range: 5–40mm). These stones were complex staghorn stones (11 cases), multiple stones (10 cases) and single stones (11 cases). Stones were present in anatomically uncomplicated kidneys in 25 cases; complex anatomy included horseshoe kidney (2 cases), calyceal diverticulum (2 cases), previously treated pelviureteric junction obstruction (2 cases) and prune belly syndrome with partial upper renal tract dysplasia (1 case).

**Table 1:** Distribution of percutaneous nephrolithotomy (PCNL) by age

Age of patient at PCNL	Number of patients undergoing PCNL
<5 years	7
5–10 years	10
11–17 years	14

In the majority of cases (26/32), a single puncture alone was required to achieve maximal stone clearance although six cases did require the placing of two punctures. No case required more than two punctures. Of the single punctures, 19 were via a lower pole calyx, 1 via an equatorial calyx and 6 via an upper pole calyx. All cases requiring multiple punctures included a lower pole puncture with either an equatorial or upper pole puncture in addition. The composition of the stones was recorded in 28 cases (Table 2).

**Table 2:** Stone composition

Stone composition	Number of stones
Magnesium ammonium phosphate	11
Calcium oxalate/phosphate	9
Calcium oxalate	4
Calcium phosphate	3
Cysteine	1

There was no significant bleeding encountered either during the operation or in the post-operative setting. No patient required a blood transfusion. Four patients developed post-operative pyrexia within the first 24 hours, requiring the continuation of parenteral antibiotics; in these patients, the removal of the pigtail nephrostomy was deferred by 24 hours, resulting in an increase in hospital stay of 24 hours. All other patients had the percutaneous access removed within 36 hours of surgery and were discharged on the second post-operative day.

Twenty-seven renal units (84%) were stone free by radiological assessment. Two patients (2 renal units) required supplementary extracorporeal lithotripsy in order to be rendered stone free (91% were therefore stone free following PCNL and ESWL) and three patients (three renal units) underwent a second PCNL. One of the patients undergoing a second PCNL was left with a small residual renal calculus for which no further treatment has been required. At 12 months of ultrasonography observation, the stone remains unaltered and asymptomatic. Of the five renal units not cleared of stones by the index procedure, three had undergone a single puncture and two a double puncture. Two of these cases were staghorn stones made up from magnesium ammonium phosphate, two were multiple stones made from calcium phosphate and one was multiple stones made from calcium oxalate.

## Discussion

In our experience, paediatric PCNL using adult instruments is a safe treatment modality and good stone clearance rates (84%) can be achieved with no major complications. This compares favourably with published series over the past 15 years (Table 3).^11–24^ Previous series have shown that PCNL can be performed safely in children with stone clearance rates of 58–93% and complication rates of 0–10%. The largest paediatric PCNL experience was reported by Samad *et al*, who reported 188 PCNLs on a population with a mean age of 6.5 years using a 22Fr tract.^20^ In this series, 90% of cases were completed using a single tract with an overall clearance rate of 76% and a complication rate of 5%. Nouralizadeh *et al* reported a 10% complication rate for patients with a mean age of 3.1 years (lowest in series), with an average stone burden of >30mm.^22^ Aron *et al* used multiple tracts and achieved a stone clearance rate of 89% with no reported complications.^19^

**Table 3:** Paediatric percutaneous nephrolithotomy (PCNL) series in the literature

Authors	Number of patients	Number of PCNLs	Mean age (years)	Stone burden (average)	Tract size (Fr)	Single tracts (%)	Stone clearance (%)	Complications (%)
Mor *et al*, 1997^11^	25	25	8	–	–	100	68	0
Jackman *et al*, 1998^12^	11	11	3.4	120mm^2^	11	100	89	0
Badawy *et al*, 1999^13^	60	60	6	–	24	100	83	3
Sahin *et al*, 2000^14^	14	16	11	301mm^2^	24–30	100	69	7
Desai *et al*, 2004^15^	56	56	10	22mm	22	40	89	0
Dawaba *et al*, 2004^16^	65	72	5.9	–	22	85	93	3
Boormans *et al*, 2005^17^	23	26	9.5	608mm^2^	18	100	58	8
Raza *et al*, 2005^18^	37	46	6.4	56mm	18	100	79	6
Aron *et al*, 2005^19^	19	19	4.2	972mm^2^	24	26	89	0
Samad *et al*, 2006^20^	169	188	6.5	26.2mm	22	90	76	5
Kapoor *et al*, 2008^21^	31	31	9.6	15mm	24–30	100	84	0
Nouralizadeh *et al*, 2009^22^	20	26	3.1	33mm	26	100	79	10
Unsal *et al*, 2010^23^	44	45	9.3	15–52mm	12–18, 24–26	90	83	22
Kumar *et al*, 2011^24^	11	12	11.7	848mm^2^	24–30	83	58	8
Present series	31	32	10.8	28mm	28	81	84	0

We experienced a low rate of complications using the same instruments and technique that we use in adult surgery. There were no bleeding complications using this access.

There are studies that have compared instrument size in PCNL and complication rates. Bilen *et al* looked at a cohort of 46 paediatric patients and compared using adult instruments via a 26Fr tract, paediatric instruments via a 20Fr tract and minimal access via 14Fr tract.^9^ They found that smaller tracts did not significantly affect stone-free rates but achieved lower transfusion rates.

Similarly, Unsal *et al* showed that in children aged 8–16, adult (*n*=15) and paediatric instruments (*n*=12) achieved near equal clearance rates of 81.3% and 83.3% respectively.^23^ In Unsal’s series, tracts to pass adult instruments were typically 24–26Fr compared with 12–18Fr for smaller instruments. They highlighted that there was more bleeding associated with larger tracts as indicated by a statistically significant mean haemoglobin drop of 2.6g/dl for larger tracts versus 1.2g/dl.

More recently, Guven *et al* compared 60 PCNLs using paediatric instrumentation with 80 performed with adult-sized instruments and looked at complications using the standardised Clavien classification.^25^ They found that the success rates were similar although post-operative haemoglobin drop was higher in the group where adult sized instruments were used. While we have not noted bleeding complications in our series, the evidence suggests that smaller tracts translate into lower transfusion rates but equivalent stone clearance rates.

The PCNL tract size does not impact on renal function. In animal models, it has been suggested that renal parenchymal damage resulting from the creation of a nephrostomy tract is small compared with overall renal volume, regardless of the size of the nephrostomy tract, and one could infer that there is no advantage to using a small access sheath based on renal scarring alone.^26^ Moreover, Mor *et al* showed that the use of a tract dilated to 24Fr or 26Fr did not lead to a significant loss of renal function on post-operative radioisotope scanning.^11^

In our series, post-operative dimercaptosuccinic acid (DMSA) imaging was not performed routinely to look for renal scarring resulting from the procedure. Some patients had further DMSA imaging at variable intervals following the procedure if they developed further urine infections. In total, pre and post-operative DMSA imaging was available in four renal units and no scarring as a result of the PCNL tract alone was discernible in those cases. In addition, no child had any discernible change in serum creatinine post-operatively although we accept this is not an accurate measure of renal function in children with a normal contralateral kidney.

The use of a larger tract in our series did not lead to a drop in renal function or bleeding problems. Provided the quality of the puncture and subsequent tract is high, there is no greater morbidity than that reported from miniperc. Large tracts and instruments can facilitate more rapid and complete stone clearance.

## Conclusions

We report our experience of PCNL in a paediatric population using access of 28Fr. We have demonstrated that the procedure can be performed safely and that excellent stone clearance rates can be attributable to the improved access provided by the bigger instruments with wider working channels. In addition, with access to flexible endoscopes and multimodal energy sources for stone destruction and removal, complete stone clearance and acceptable stone recurrence rates can be achieved in most patients.

We believe the use of adult size tracts with standard PCNL nephroscopes facilitates stone clearance with no increase in morbidity. Small scopes with smaller working channels can restrict the surgeon in the ability to clear fragments. Furthermore, most patients want to avoid multiple procedures with residual fragments. Morbidity probably relates more to the quality and accuracy of the tract rather than the tract size. We confirm that standard adult PCNL techniques are safe and effective in the paediatric setting.
